# Does insular cortex lesion cause tinnitus in rats?

**DOI:** 10.22038/IJBMS.2022.63698.14083

**Published:** 2022-10

**Authors:** Minoo Karimi, Saeid Farahani, Farinaz Nasirinezhad, Shohreh Jalaei, Helnaz Mokrian, Ali Shahbazi

**Affiliations:** 1 Department of Audiology, School of Rehabilitation, Tehran University of Medical Science, Tehran, Iran; 2 Physiology Research Center, Faculty of Medicine, Iran University of Medical Sciences, Tehran, Iran; 3 Department of Physiology, Faculty of Medicine, Iran University of Medical Sciences, Tehran, Iran; 4 Department of Physiotherapy, School of Rehabilitation, Tehran University of Medical Sciences, Tehran, Iran; 5 Cellular and Molecular Research Center, Iran University of Medical Sciences, Tehran, Iran; 6 Department of Neuroscience, Faculty of Advanced Technologies in Medicine, Iran University of Medical Sciences, Tehran, Iran

**Keywords:** Insular cortex, Prepulse inhibition, Rats, Startle reflex, Tinnitus

## Abstract

**Objective(s):**

Tinnitus is defined as ringing of the ears that is experienced when there is no external sound source, and is an auditory phantom sensation. The insula as a multimodal cortex has been shown to be involved in the processing of auditory stimuli rather than other sensory and motor processing and reported to correlate with some aspects of tinnitus. However, its exact role is not clear. The present study aimed to investigate the effect of excitotoxic lesions limited to the insular cortex on the ability to detect a gap in background noise.

**Materials and Methods:**

Gap detection test and prepulse inhibition, two objective measurements of auditory startle response, were measured, in 33 male Wistar rats, before and up to four weeks after insular lesion in three experimental groups (sham, control, and lesion).

**Results:**

The ability to detect the gap interposed between 60 db background noise was impaired at weeks 2, 3, and 4 following insular lesion, while prepulse inhibition remained intact up to four weeks after surgery.

**Conclusion:**

These findings indicated that excitotoxic lesions of the insular cortex may produce a tinnitus-like phenomenon in rats while sparing the hearing sensitivity; suggesting that the insular cortex may have a role in the development of tinnitus.

## Introduction

Tinnitus is defined as subjective sound perception in the absence of an external sound source(1). Tinnitus may cause emotional and psychological distress, which is not proportional to the severity of tinnitus (2). The prevalence of tinnitus is approximately 12–30% worldwide (3), and about 1–2% of patients experience severe tinnitus with impaired quality of life (4).

The pathophysiological mechanism of tinnitus includes impairment in one or more areas of the peripheral or central auditory pathways, with reported abnormal spontaneous neural activity or reorganization of the pathways (5-7). Recent studies suggest that in addition to auditory-related areas, non-auditory areas of the brain are also involved in tinnitus disorder (5, 8-12). Possible cortical generators of tinnitus include parahippocampal, anterior cingulate, dorsolateral prefrontal, insular, supplementary motor, and orbitofrontal cortices (13). There is no clear explanation of how these areas function in tinnitus, however, studies reported their involvement in establishment of auditory memory of tinnitus, allocated attention to tinnitus, integrating cognition and emotion related to tinnitus, and conscious perception of tinnitus (13, 14).

The insular lobe, Brodmann areas 13 through 16 (15), is a multimodal cortical area that functions in interoception, somatosensory, gustation, pain, olfaction, speech, and emotional processing (16, 17). Furthermore, lesion and imaging studies indicated that the insular cortex is involved in auditory processing as well (18-20). However, in most of these studies, the lesion was not limited to the insular cortex, and reported results may include the role of other affected brain areas. Some neurons of the insular cortex respond directly to auditory stimuli, and it has been shown that the insular cortex has a direct connection with the auditory cortex and medial geniculate bodies (21, 22), indicating the role of the insula in auditory or related processing (23, 24). Electrical stimulation of the insular cortex will produce the sensation of buzz or whistle (17, 25). Additionally, neuroimaging studies revealed the insular abnormal activity in tinnitus patients before and during treatment, and relevance of insular activity in maintaining tinnitus has been determined. The insular cortex has been suggested as the final common pathway in the neural underpinning of tinnitus (26). 

Increase in gamma-band connectivity between the insula with primary and secondary auditory cortices in tinnitus patients has been suggested to be related to increased emotional response and/or adaptation to the tinnitus sound perception (27, 28). The insula with other brain areas may participate in a distress-related network in tinnitus, pain (29), and other unpleasant somatosensory experiences (30). Accordingly, negative relationship between the volume of gray matter in the insula and distress related to tinnitus (31) as well as positive correlation between cortical thickness of the anterior insula and tinnitus distress was reported (32). Additionally, the role of the insula in the regulation of the autonomous system via sympathetic nuclei output has been shown in tinnitus distress (13). On the other hand, tinnitus similar to neuropathic pain, with respect to phantom sensations, may have common pathophysiological mechanisms (33). Nevertheless, a few studies reported that insular lesions can result in central pain (34). Furthermore, the activity of the insular cortex and anterior cingulate cortex as the main nodes of the salience network (35), has been reported to improve the auditory threshold possibly by allocating the attention and importance to external auditory stimuli and probably to internally generated sounds such as tinnitus (13, 36). Accordingly, it has been reported that the activity of insula and cingulate cortices modify the activity in auditory cortices in tinnitus patients (37).

Although the role of the insula in the emotional aspect of tinnitus as a negative and unpleasant emotional experience is partly understood, the role of the insula in the development of tinnitus is not clear. In fact, there is no study to report that defection of the insula can be related to producing tinnitus. The present study aimed to investigate whether the excitotoxic lesion of the insular cortex can produce tinnitus in rats.

## Materials and Methods

***Animals***

A number of 33 adult male Wistar rats weighing 175–250 gr were used in this study. Animals were purchased from Pasteur Institute, Tehran, Iran and transferred to the Center for Experimental and Comparative Study, Iran University of Medical Sciences, Tehran, Iran. Rats were housed in groups of two/three in cages and maintained on a 12 hr light /12 hr dark cycle schedule (lights on at 7 A.M.) and behavioral tests were carried out on the light phase. Animals had *ad libitum* access to food and water. One week after arrival, rats were assigned randomly to three groups; control, surgical sham, and lesion groups (n:10-12/group). All procedures were carried out in accordance with the National Institutes of Health guide regarding the care and use of laboratory animals (NIH Publications No. 8023, revised 1978), and were approved by The Research Ethical Committee at Tehran University of Medical Science, Iran.

***Surgery***

Surgeries were performed after chloral hydrate anesthesia (450 mg/kg, intraperitoneally (IP))(38) and were placed in a stereotaxy apparatus (Stoelting, USA), then the skull was exposed. To prevent tympanic membrane perforation during head fixation, non-puncture ear bars were used. The needle of a Hamilton syringe lowered into the target area and 0.6ul NMDA solution in PBS (10 mg/ml) was bilaterally infused into the insular cortex at a rate of 0.2 ul/min (39, 40). The needle was left in place for another 1 min to facilitate diffusion of the injection volume. Sham-lesioned control animals had similar surgical procedures but without NMDA injection. The coordinates of the injection site based on the Paxinos atlas for rats to target insular cortex were: Anterior-Posterior -1 mm and Medial–lateral 5.8 mm relative to Bregma. The injection needle was lowered 7.5 mm with an angle of 12 degrees (41). The recovery period after stereotaxic surgery included 7 days (42).

***Acoustic startle apparatus and behavioral testing***

Gap detection and PPI tests were measured in the SR-LAB system (San Diego Instruments, San Diego, CA, USA). The chamber contained a Plexiglas cylinder (8.8 cm inside diameter, 18.4 cm inside length) positioned in a ventilated sound-attenuated enclosure. Animal movement (force) is converted to voltage (microvolts) by a piezoelectric transducer beneath the Plexiglas cylinder. Broadband noise burst (20 ms duration, 0.1 ms rise and fall time) was delivered to the chamber with a loudspeaker mounted 24 cm above it. The loudspeaker output was calibrated with a sound level meter (Bruel-Kjaer 2230, Denmark).

Gap detection and PPI sessions started with 2 and 2.5 min acclimation periods, respectively, consisting of background broad-band noise (60 and 55 db SPL, respectively) that continued throughout the sessions.

The gap detection session consisted of 12 without gap trials and 12 gap trials (24 trials). Without gap trial consisted of an abrupt broadband noise burst (20 ms duration, 115 db SPL) and the gap trial consisted of a brief gap (50 ms duration) started 100 ms before the abrupt broadband noise burst (20 ms duration, 115 db SPL). The acclimation period was followed by two without-gap trials for habituation of animal reflexes that were removed from the analysis for a more stable baseline. 22 remaining trials presented in a pseudorandomized order with an inter-trial interval of 15–20 sec (43). 

The PPI session consisted of 45 startle trials comprising 10 startle-alone trials (100 db SPL broadband noise, 40 ms duration), 15 prepulse alone trials (70 db SPL, 75 db SPL, and 80 db SPL; 20 ms duration: 5 of each intensity), 15 startle trials preceded by a prepulse (PP) with different intensities, and 5 no-stimulus trials. After an acclimation period, 5 startle-alone trials were presented to habituate the startle response and removed from the analysis. 40 remaining trials were presented in a pseudorandomized order with an inter-trial interval of 15–20 sec (44). 

Startle recording time in both gap detection and PPI tests started with the initiation of each trial, and continued to 100 ms after auditory stimulus delivery, except in no-stimulus trials where the recording was carried out without stimulus delivery. The maximum peak of the startle response in each 100ms recording window was considered in data analysis.

Gap detection tests were carried out at baseline (week0) and one, two, three, and four weeks after surgery (week1–4). PPI was carried out before surgery (week0), one week (week1), and four weeks (week4) with the aim to rule out sensory hearing loss and possible deficit in the central processing of auditory startle reflex (45-48).

***Histology***

At the end of behavioral tests, rats were overdosed with pentobarbital sodium (100 mg/kg, IP) and perfused transcardially with 3.7% paraformaldehyde in normal saline. Extracted brains were placed in 3.7% paraformaldehyde for about 10 days, then processed by an automated tissue processor (LEICA TP1020). Coronal slices (10 μm thick) were taken from Bregma -1.5 mm to -0.2 mm using a rotary microtome and mounted on the gelatin-coated slides. For evaluation of lesion sites, cresyl violet staining was used. Injection sites are depicted in Figure 3.

***Statistical analysis***

For data analysis of the gap detection test, means of without gap trials and with gap trials were used, then relative startle response (percentage of the startle response in without gap trials) was calculated: Mean of with gap trials×100/ Mean of without gap trials. For PPI data analysis, the mean of startle-alone trials and the average of prepulse trials (70, 75, and 80 db) were used to compute the percentage of PPI: Mean of prepulse trial averages ×100/ Mean of startle-alone trials. Analysis was carried out with SPSS Ver. 20, and two-way repeated measure analyses of variance (ANOVA) were used: session was considered as within-subject factor and experimental group as between-subject factor. Tukey *post hoc* analysis was used for multiple comparisons. Mean differences with a *P*-value less than 0.05 were considered significant.

## Results

***Effect of insular cortex lesion on gap detection***

**Figure 1:**
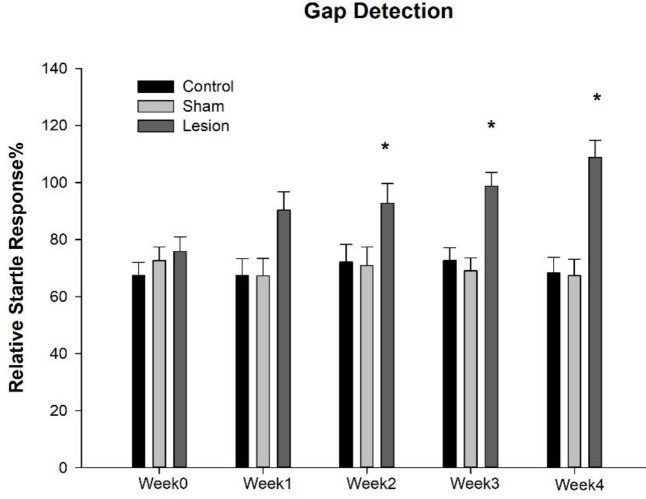
Mean (± SEM) of relative startle response of rats (gap detection) at different sessions before (week0) and after intervention (week1–4). The lesion group had an insular cortex lesion bilaterally. * *P*<0.05, significant difference compared with control and sham groups at the same session (week)

The mean (± SEM) of the relative startle response (gap detection) of control, sham, and lesion groups was presented in Table 1 and Figure 1. Analysis revealed the significant effect of session×group [F(8,120)=2.522, *P*=0.014]. Bonferroni *post hoc* analysis showed a significant difference in relative startle response two weeks after surgery in the lesion group compared with control (week2: *P*=0.044; week3: *P*=0.001; and week4: *P*=0.0001) and sham (week2: *P*=0.037; week3: *P*=0.0001; and week4: *P*=0.0001) groups.

***Effect of insular cortex lesion on PPI Test***

**Figure 2:**
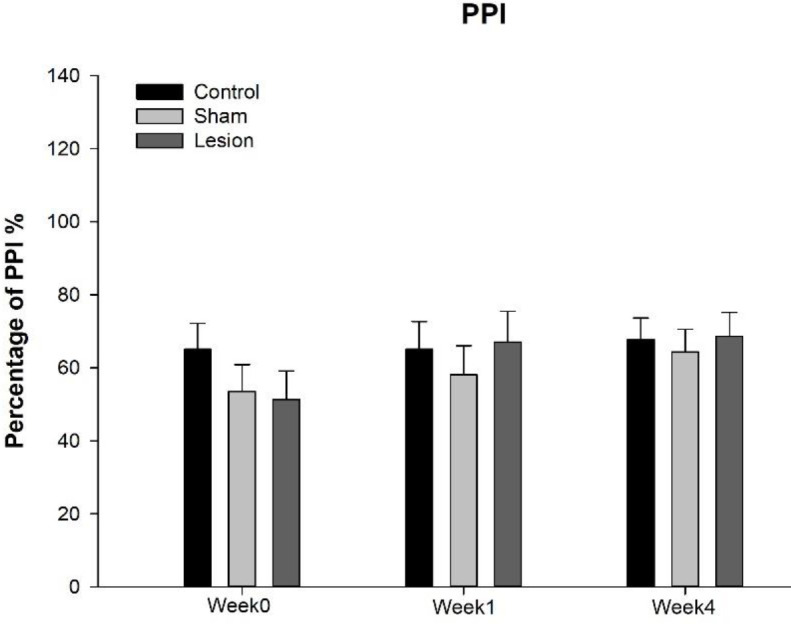
Mean (± SEM) of PPI percentage of rats at different sessions before (week0) and after intervention (week1&4). The lesion group had an insular cortex lesion bilaterally

The mean (± SEM) of PPI percentage in experimental groups was presented in Table 2 and Figure 2. To assess the effect of insular cortex lesion on the PPI test, means of percentage of PP in all intensities were calculated, and two way ANOVA was carried out; the result demonstrate no significant effect on session×group [F(4,60)=1.270, *P*=0.292], and Bonferroni *post hoc* test revealed no significant difference between groups in any sessions.

**Table 1:** Mean (± SEM) of relative startle response of rats (gap detection) at different sessions before (week0) and after intervention (week1–4). The lesion group had an insular cortex lesion bilaterally

	Pre-surgery	Post-surgery			
Group	**Week0**	**Week1**	**Week2**	**Week3**	**Week4**
Control	67.41±4.63	67.41±5.87	72.12±6.26	72.68±4.41	68.34±5.52
Sham	72.61±4.84	67.25±6.13	70.91±6.54	69.07±4.61	67.37±5.76
Lesion	75.87±5.07	90.38±6.43	92.78±6.86*	98.78±4.84*	108.82±6.05*

* *P*<0.05, significant difference compared with control and sham groups at the same session (week)

**Table 2:** The mean (± SEM) of PPI percentage of rats at different sessions before (week0) and after intervention (week1&4). The lesion group had an insular cortex lesion bilaterally

	Pre-surgery	Post-surgery	
Group	**Week0**	**Week1**	**Week4**
Control	65.11±7.09	65.11±7.60	67.73±5.92
Sham	53.74±7.40	58.10±7.94	64.35±6.19
Lesion	51.30±7.77	67.11±8.33	68.70±6.49

**Figure 3:**
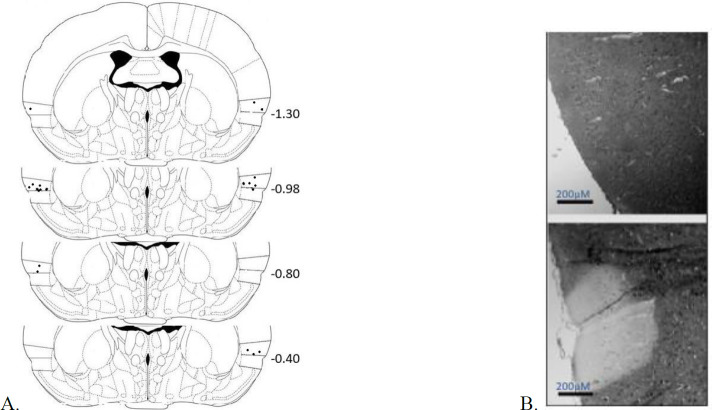
Histologic verification of lesion of the insular cortex in experimental rats. The location of the lesion center in the insular cortex based on the Paxinos atlas. A/P coordinates (in mm) are given relative to Bregma, (n: 10-12/group). B: samples of sections stained with cresyl violet in the control (above) and the insular lesion groups (below). Scale bar is equal to 200 µM

## Discussion

Tinnitus, defined as ringing of the ears experienced when there is no external sound source, is an auditory phantom sensation (49). In the present study, to investigate the role of insular cortex lesions in developing tinnitus in rats, acoustic startle responses to 115 db with/without a gap interposed in the 60 db background noise -gap detection - were measured. In addition, to evaluate normal hearing sensitivity, PPI tests were carried out. Finally, the location of the excitotoxic lesion was verified by cresyl violet staining of prepared brain sections.

Gap detection data after insular lesions demonstrated a decrease in relative startle response. Decreases in relative startle response or gap detection deficit have been used as objective indicators of tinnitus in rodent studies (43, 50). In the present study, deficit in gap detection was evident two weeks after the insular lesion and preserved up to four weeks after surgery in the lesion group, indicating tinnitus initiation two weeks after insular lesion and may persist at least up to four weeks. These findings propose that tinnitus induced by insular lesions is not a short-term deficit. Some studies have used the PPI test as a tool for screening hearing sensitivity (45-48). Here, PPI data showed no significant alteration in the percentage of PPI after insular lesion in any of the time measurements, suggesting a normal hearing sensitivity and intact central auditory processing, which preclude the role of possible hearing loss in tinnitus induced by insular cortex lesion.

The role of different non-auditory brain regions in tinnitus or related distress has been reported in many studies. Accordingly, the insular cortex has been proposed as one of the cortical areas which are involved in both the perceptual and emotional aspects of tinnitus (51). Neuroimaging and lesion studies have shown that the insular cortex is involved in auditory processing (18-20). Additionally, single-cell recordings in primates demonstrated that specific neurons in the insula directly respond to auditory stimuli (23, 24). Furthermore, electrical stimulation of the insular cortex has been reported to induce auditory responses such as hallucinations of sounds or tinnitus (17, 25, 52). Recent study appropriately mapped the auditory functions of the insular cortex. It has shown that the posterior granular/dysgranular sector of the insular cortex is highly connected to primary and secondary sensory cortices including the auditory cortex, and functionally is related to perception. Accordingly, electrical stimulation of the posterior insula produces perceptual auditory sensations in epileptic patients undergone medical surgery (51). On the other hand, the same study revealed that the anterior insula is highly connected to limbic and cognitive cortices of the prefrontal cortex, implying the role of the anterior insula in emotional and higher cortical processing. These findings have proposed the possible role of the insula in both perceptual and emotional aspects of auditory processing. 

Furthermore, structural and functional studies have shown the reduction in insular gray matter volume, structural alterations of the insular cortex, and sympathetic hyperactivity that were related to distress in tinnitus patients (31, 32). It has been reported that insular cortex activity is altered in tinnitus or during treatment, and the insula was suggested as a final common pathway for tinnitus (26). The role of the insula in maintaining tinnitus and related distress has been reported in many studies, but its role in developing tinnitus has remained to elucidate. 

The findings of the present study suggest the role of the insula in developing tinnitus in rats. How insular cortex lesion may produce tinnitus is not clear, but considering the connections of the insular cortex to auditory areas in the temporal lobe and thalamic medial geniculate bodies (21, 22), and reciprocally connections to tinnitus-related cortical areas such as medial and orbitofrontal prefrontal cortices (33), suggest involvement of insula in a possible cortical network of tinnitus. 

In addition to cortical areas, the insula has considerable connections with subcortical and brain stem nuclei (21, 53-56) which may be implicated in tinnitus perception and emotion. One of the proposed mechanisms is the attentional network with cortical and subcortical components such as cingulate and brain stem nuclei. The insula, as well as the dorsal anterior cingulate cortex, are two major components of the salience network (35), by which significant events and important inputs will detect and will result in appropriate responses, this network inhibits inappropriate stimuli as well. Considering the tinnitus-related sounds as salience internal sounds (26), it is hypothesized that insular lesions may impair the inhibition of phantom sounds or irrelevant auditory stimuli. 

Another possible mechanism by which the insula may be involved in tinnitus is gating mechanisms in addition to the top-down control of attentional networks (57, 58). Sensory gating as a protective mechanism has been investigated in many studies in humans and rodents. This pre-attentional mechanism inhibits the irrelevant and noise stimuli from lower sensory nuclei to enter the higher sensory processing (11). Disturbances in sensory gating have been reported in tinnitus patients as well, although the reports are not consistent (59). Disturbances in sensory gating may permit the noises to enter the higher cortical areas and produce phantom sensations in addition to sensory overload. Insula via massive connections with cortical and subcortical regions may be involved in gating of external and/or abnormal internal auditory stimuli. Accordingly, It has been shown that insular lesion affects the corticofugal pathway and results in alteration in cochlear activity (60). 

## Conclusion

Following excitotoxic lesions of the insular cortex, a decrease in relative startle response or gap detection deficit has been observed, suggesting development of tinnitus in rats. These findings support our hypothesis that the insular cortex, in addition to proposed involvement in maintaining tinnitus and related distress, may also be involved in development of tinnitus, probably via top-down inhibitory mechanisms. Furthermore, possible hearing loss assessed by the PPI test, as the common etiology of tinnitus, was not evident in insular lesion rats. Finally, it suggested that possible inhibitory or gating functions of the insula should be considered in pathophysiological mechanisms of tinnitus in addition to involvement in the distress accompanying tinnitus. 

## Authors’ Contributions

MK and AS Designed the experiments; AS, MK, and HM Performed experiments and collected data; AS, MK, and SJ Analyzed and Interpreted the Results; AS and SF Supervised, directed, and managed the study; MK and FN Edited the article; MK, AS, SF, FN, SJ, and HM Approved the final version of the manuscript to be published .

## Conflicts of Interest

None.
